# Clinical Results of Bristow and Latarjet Procedures in Patients With Anterior Shoulder Instability: A Systematic Review and Meta-Analysis

**DOI:** 10.7759/cureus.92782

**Published:** 2025-09-20

**Authors:** Masaki Karasuyama, Junichi Kawakami, Shuhei Yamamoto, Takashi Ariie, Takashi Tsuruta, Tomohiko Minamikawa, Hiroki Ohzono, Hiroaki Moriyama, Masafumi Gotoh

**Affiliations:** 1 Department of Rehabilitation, Minamikawa Orthopedic Hospital, Fukuoka, JPN; 2 Department of Anatomy, The Nippon Dental University School of Life Dentistry at Niigata, Niigata, JPN; 3 Department of Rehabilitation, Shinshu University Hospital, Matsumoto, JPN; 4 Department of Physical Therapy, School of Health Sciences at Fukuoka, International University of Health and Welfare, Fukuoka, JPN; 5 Department of Orthopedic Surgery, Minamikawa Orthopedic Hospital, Fukuoka, JPN; 6 Department of Orthopedic Surgery, Kurume University Medical Center, Kurume, JPN

**Keywords:** anterior shoulder instability, bristow procedure, latarjet procedure, meta-analysis, systematic review

## Abstract

Anterior shoulder instability poses a significant challenge, particularly in active populations. The Bristow and Latarjet surgical approaches address this issue; however, their comparative efficacy remains debated. This study conducted a meta-analysis of randomized controlled trials and observational studies to assess the efficacy of the Bristow and Latarjet procedures in patients with anterior shoulder instability.

We searched electronic databases, including CENTRAL, MEDLINE, and EMBASE, for both randomized controlled trials and observational studies from inception to May 2023. To ensure the inclusion of recent evidence, we conducted an updated search in March 2025 to identify studies published between May 2023 and March 2025. We included studies that recruited patients with anterior shoulder instability and compared the Bristow and Latarjet procedures. The primary outcomes included redislocation rates, instability symptoms, and the Rowe score. The secondary outcomes included return to sports, bone nonunion, bone resorption, and operative complications. The methodological quality of included studies was assessed using the RoB 2 (Risk of Bias 2) tool for randomized controlled trials and the ROBINS-I (Risk of Bias in Non-randomized Studies of Interventions) tool for nonrandomized studies. Meta-analysis using random-effects models was performed to determine the significance of the pooled results.

The database search yielded 3,115 records, four of which met the inclusion criteria, comprising a total of 354 patients with 383 shoulders (211 shoulders treated with the Bristow technique and 172 with the Latarjet technique). For the primary outcome of redislocation, all the studies included in this review reported no events in any patient who underwent either the Bristow or Latarjet procedure. In addition, there was no significant difference in instability symptoms between the Bristow and Latarjet procedures (risk ratio (RR), 0.66; 95% confidence interval (CI), 0.16-2.66; P = 0.55). The Rowe score showed a significant difference between the Bristow and Latarjet procedures (mean difference, 1.73; 95% CI, 0.38-3.07; P = 0.01); however, the observed difference did not reach the minimal clinically important difference. Among the secondary outcomes, there were no significant differences between the two procedures in return to sports (RR, 1.00; 95% CI, 0.94-1.07; P = 0.93), bone nonunion (RR, 2.09; 95% CI, 0.94-4.62; P = 0.07), or complications (RR, 1.06; 95% CI, 0.45-2.49; P = 0.90). Three studies used different methods and definitions for evaluating bone resorption; therefore, we did not analyze it.

There is currently insufficient evidence to conclude a difference in postoperative outcomes between the Bristow and Latarjet procedures, primarily due to the limited number of included studies, which reduces statistical power and affects the precision of effect estimates. Further high-quality randomized controlled trials focusing on symptoms of instability are needed to inform appropriate procedure selection.

## Introduction and background

Anterior shoulder instability (ASI) predominantly occurs in active populations, with approximately 95% of cases involving the anterior direction of instability [1]. ASI occurs due to disruption of the static and dynamic stabilization mechanisms of the glenohumeral joint, and in some instances, pain [2]. The incidence of ASI, as reported in studies targeting the general population of the United States, is approximately 0.08 individuals per 1000 persons per year [3]. Notably, in males under 30 years of age, a higher incidence of 1.69 individuals per 1000 persons per year is observed [4]. Athletes engaged in sports activities exhibit an incidence rate over twice as high as those participating in recreational activities, signifying an elevated risk of developing ASI within more active populations [5].

Arthroscopic Bankart repair is the most commonly performed surgical treatment for ASI; however, rugby and American football players exhibit elevated redislocation rates [6]. For patients with a heightened risk of redislocation, the Bristow-Latarjet procedure, involving the transfer of the coracoid process to the anterior rim of the glenoid, is employed. The Bristow procedure positions the excised coracoid process in an upright orientation directly below the glenoid, secured with a single screw [7]. In contrast, the Latarjet procedure places the coracoid process in a laid-down orientation after osteotomy at its base, fixing it with two screws to the anterior glenoid, which provides broader bony contact and a larger sling effect for stabilization [8]. Both procedures can be performed using open or arthroscopic approaches. In recent years, arthroscopic techniques have been increasingly adopted for both the Bristow and Latarjet procedures, with reported advantages in visualization and graft positioning.

In 2019, a systematic review was published encompassing 41 studies utilizing the Bristow procedure and 18 studies of the Latarjet procedure in patients with ASI [9]. While this review suggested that the Bristow procedure might lead to a reduced recurrence rate compared with the Latarjet procedure, it lacked randomized controlled trials (RCTs) and observational studies directly comparing these two surgical techniques, precluding the feasibility of conducting a meta-analysis.

In 2021, the first randomized controlled trial comparing the Bristow and Latarjet procedures for ASI was published [10]. Subsequently, several observational studies on these two techniques have been published, contributing to an increasing body of evidence regarding the efficacy of either method for treating ASI [11,12]. By aggregating and analyzing the contents of these studies, further insights could be gained concerning the comparison of postoperative outcomes between the Bristow and Latarjet procedures in patients with traumatic ASI. Therefore, the objective of this study was to elucidate the efficacy of the Bristow and Latarjet procedures in patients with ASI through a meta-analysis of RCTs and observational studies, with the primary research question being whether either technique results in superior clinical outcomes.

## Review

Material and methods

This study was conducted in accordance with the Preferred Reporting Items for Systematic Review and Meta-Analyses (PRISMA) 2020 (Table 1) [13]. The protocol for this study was registered in the Open Science Framework (https://osf.io/vfwgu/).

**Table 1:** PRISMA 2020 checklist The PRISMA 2020 checklist is reproduced with permission under a Creative Commons Attribution License from Page et al. [13]. Available at http://www.prisma-statement.org/ PRISMA: Preferred Reporting Items for Systematic Review and Meta-Analyses

Section and topic	Item #	Checklist item	Location where item is reported
TITLE			
Title	1	Identify the report as a systematic review.	Title Page
ABSTRACT			
Abstract	2	See the PRISMA 2020 for Abstracts checklist.	Abstract
INTRODUCTION			
Rationale	3	Describe the rationale for the review in the context of existing knowledge.	Introduction
Objectives	4	Provide an explicit statement of the objective(s) or question(s) the review addresses.	Introduction
METHODS			
Eligibility criteria	5	Specify the inclusion and exclusion criteria for the review and how studies were grouped for the syntheses.	Methods
Information sources	6	Specify all databases, registers, websites, organisations, reference lists and other sources searched or consulted to identify studies. Specify the date when each source was last searched or consulted.	Methods
Search strategy	7	Present the full search strategies for all databases, registers and websites, including any filters and limits used.	Methods
Selection process	8	Specify the methods used to decide whether a study met the inclusion criteria of the review, including how many reviewers screened each record and each report retrieved, whether they worked independently, and if applicable, details of automation tools used in the process.	Methods
Data collection process	9	Specify the methods used to collect data from reports, including how many reviewers collected data from each report, whether they worked independently, any processes for obtaining or confirming data from study investigators, and if applicable, details of automation tools used in the process.	Methods
Data items	10a	List and define all outcomes for which data were sought. Specify whether all results that were compatible with each outcome domain in each study were sought (e.g. for all measures, time points, analyses), and if not, the methods used to decide which results to collect.	Methods
	10b	List and define all other variables for which data were sought (e.g. participant and intervention characteristics, funding sources). Describe any assumptions made about any missing or unclear information.	Not applicable
Study risk of bias assessment	11	Specify the methods used to assess risk of bias in the included studies, including details of the tool(s) used, how many reviewers assessed each study and whether they worked independently, and if applicable, details of automation tools used in the process.	Methods
Effect measures	12	Specify for each outcome the effect measure(s) (e.g. risk ratio, mean difference) used in the synthesis or presentation of results.	Methods
Synthesis methods	13a	Describe the processes used to decide which studies were eligible for each synthesis (e.g. tabulating the study intervention characteristics and comparing against the planned groups for each synthesis (item #5)).	Methods
	13b	Describe any methods required to prepare the data for presentation or synthesis, such as handling of missing summary statistics, or data conversions.	Methods
	13c	Describe any methods used to tabulate or visually display results of individual studies and syntheses.	Methods
	13d	Describe any methods used to synthesize results and provide a rationale for the choice(s). If meta-analysis was performed, describe the model(s), method(s) to identify the presence and extent of statistical heterogeneity, and software package(s) used.	Methods
	13e	Describe any methods used to explore possible causes of heterogeneity among study results (e.g. subgroup analysis, meta-regression).	Methods
	13f	Describe any sensitivity analyses conducted to assess robustness of the synthesized results.	Methods
Reporting bias assessment	14	Describe any methods used to assess risk of bias due to missing results in a synthesis (arising from reporting biases).	Methods
Certainty assessment	15	Describe any methods used to assess certainty (or confidence) in the body of evidence for an outcome.	Methods
RESULTS			
Study selection	16a	Describe the results of the search and selection process, from the number of records identified in the search to the number of studies included in the review, ideally using a flow diagram.	Results
	16b	Cite studies that might appear to meet the inclusion criteria, but which were excluded, and explain why they were excluded.	Results
Study characteristics	17	Cite each included study and present its characteristics.	Results
Risk of bias in studies	18	Present assessments of risk of bias for each included study.	Results
Results of individual studies	19	For all outcomes, present, for each study: (a) summary statistics for each group (where appropriate) and (b) an effect estimate and its precision (e.g. confidence/credible interval), ideally using structured tables or plots.	Results
Results of syntheses	20a	For each synthesis, briefly summarise the characteristics and risk of bias among contributing studies.	Results
	20b	Present results of all statistical syntheses conducted. If meta-analysis was done, present for each the summary estimate and its precision (e.g. confidence/credible interval) and measures of statistical heterogeneity. If comparing groups, describe the direction of the effect.	Results
	20c	Present results of all investigations of possible causes of heterogeneity among study results.	Results
	20d	Present results of all sensitivity analyses conducted to assess the robustness of the synthesized results.	Results
Reporting biases	21	Present assessments of risk of bias due to missing results (arising from reporting biases) for each synthesis assessed.	Results
Certainty of evidence	22	Present assessments of certainty (or confidence) in the body of evidence for each outcome assessed.	Results
DISCUSSION			
Discussion	23a	Provide a general interpretation of the results in the context of other evidence.	Discussion
	23b	Discuss any limitations of the evidence included in the review.	Discussion
	23c	Discuss any limitations of the review processes used.	Discussion
	23d	Discuss implications of the results for practice, policy, and future research.	Discussion
OTHER INFORMATION			
Registration and protocol	24a	Provide registration information for the review, including register name and registration number, or state that the review was not registered.	Methods
	24b	Indicate where the review protocol can be accessed, or state that a protocol was not prepared.	Methods
	24c	Describe and explain any amendments to information provided at registration or in the protocol.	Methods
Support	25	Describe sources of financial or non-financial support for the review, and the role of the funders or sponsors in the review.	Title page
Competing interests	26	Declare any competing interests of review authors.	Title page
Availability of data, code and other materials	27	Report which of the following are publicly available and where they can be found: template data collection forms; data extracted from included studies; data used for all analyses; analytic code; any other materials used in the review.	Not applicable

Study Identification

**Figure 1:**
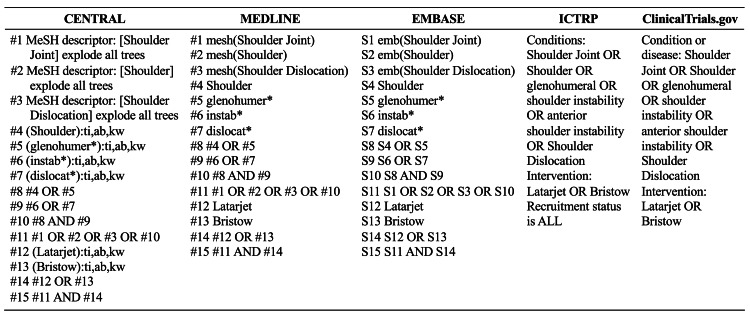
Search strategy * indicates truncation (e.g., glenohumer* retrieves glenohumeral, glenohumerus, etc.)

We searched the Cochrane Central Register of Controlled Trials (CENTRAL), MEDLINE, and EMBASE electronic databases with no limitations concerning the year of publication or language. The initial search included studies from database inception to May 2023. Since approximately one year had passed since the initial search and to capture newly published data before finalizing the review, we conducted an updated search in March 2025, covering studies published between May 2023 and March 2025. Search strategies were adjusted to meet the specifications of the individual databases (Figure 1). In addition, we checked the reference lists of eligible studies and articles that cited the eligible studies. We searched the World Health Organization International Clinical Trials Platform Search Portal (ICTRP) and ClinicalTrials.gov databases to identify ongoing clinical trials. Two independent reviewers screened the titles and abstracts and assessed their eligibility based on the full text. Disagreements between the two reviewers were resolved through discussion, and if this failed, a third reviewer acted as an arbiter.

Eligibility Criteria

We included studies that recruited patients with ASI and compared the Bristow and Latarjet procedures. Although RCTs have the most rigorous design for assessing the efficacy of intervention studies, most of the available literature on this topic consists of non-RCTs or observational studies. Therefore, we included RCTs, quasi-randomized controlled trials, and observational studies (cohort, case-control, and cross-sectional studies). We did not apply language or country restrictions. We included all the papers, including published and unpublished articles, conference abstracts, and letters. Literature reviews, expert opinions, nonhuman or cadaver studies, case reports, and clinical studies that did not evaluate the Bristow and Latarjet procedures were excluded.

Assessment of Risk of Bias

Two reviewers independently evaluated the risk of bias using the Cochrane Collaboration's Risk of Bias 2 (RoB 2) tool for RCTs [14]. We summarized the risk of bias judgments for each outcome across the different studies for the five domains: (1) the randomization process, (2) deviations from intended interventions, (3) missing outcome data, (4) measurement of the outcome, and (5) selection of the reported result. We used the signaling questions in RoB 2 and rated each domain as having a low risk of bias, some concerns, or high risk of bias. The overall risk of bias for each outcome reflected the least favorable assessment across these domains. An outcome was judged to be at low risk of bias if all domains were rated as low risk. We assessed an outcome as having some concerns if at least one domain was rated as having some concerns and none were rated as high risk. An outcome was classified as high risk of bias if one or more domains were rated as high risk or if multiple domains were rated as having some concerns.

We evaluated the nonrandomized studies using the Risk of Bias in Non-randomized Studies of Interventions (ROBINS-I) tool [15], which assesses seven domains: (1) confounding, (2) selection of participants, (3) classification of interventions, (4) deviations from intended interventions, (5) missing data, (6) measurement of outcomes, and (7) selection of reported results. Each domain and the overall risk of bias are judged as low, moderate, serious, or critical. Disagreements between the two reviewers were discussed; if this failed, a third reviewer acted as an arbiter, if necessary. Disagreements between the two reviewers were discussed; if this failed, a third reviewer acted as an arbiter, if necessary.

Types of Outcome Measures

The primary outcomes were redislocation rates, instability symptoms (including subluxations or positive symptoms of instability, such as an apprehension test), and the Rowe score [9]. The secondary outcomes included return to sports, bone nonunion, bone resorption, and operative complications [16].

Data Synthesis

We pooled the relative risk ratios (RRs) for binary outcomes and mean differences (MDs) for continuous outcomes. Where standard deviations (SD) were missing, which is a necessary component for statistical calculations in meta-analyses, we contacted the corresponding authors for details on the clinical results. If the author named in the report could not be contacted or did not respond, we calculated SDs from standard errors (SEs), 95% confidence intervals (CIs), or P values. For two studies, the SDs for the Rowe score were not available; therefore, we calculated them using a validated estimation formula [11,12]. Specifically, we derived the t statistic from the reported P value and sample sizes, calculated the SE of the MD, and then obtained the pooled within-group SD. The potential influence of using imputed data was examined through sensitivity analysis by excluding these studies from the pooled analysis.

We used the I2 statistic to measure the heterogeneity among the trials in each analysis. We interpreted the I2 statistic as follows: 0%-40% may represent insignificant heterogeneity, 30%-60% may represent moderate heterogeneity, 50%-90% may represent substantial heterogeneity, and 75%-100% may represent considerable heterogeneity.

Meta-analyses were conducted using the Cochrane Collaboration statistical software Review Manager 5.4 (The Cochrane Collaboration, London, England, UK). A random‐effects model was applied for all outcomes, as we anticipated clinical heterogeneity across studies due to differences in sample size, participant characteristics, and surgical procedures. Continuous data are expressed as mean differences and 95% CIs. For dichotomous outcomes, risk ratios (RRs) were calculated with 95% CIs. An effect was considered significant when the P value was < 0.05.

We planned to assess the potential publication bias using funnel plots and the Egger test if more than 10 trials were found. If data were available, we planned to perform a subgroup analysis for major outcomes between collision athletes vs. non-collision athletes. In addition, we planned the following sensitivity analyses for the primary outcomes to assess whether the results of the review were robust to the decisions made during the review process: (1) excluding studies that we judge as “high-risk” for bias, defined as those with a high risk in the domain of the randomization process; (2) excluding studies using imputation statistics.

Assessment of the Certainty of the Evidence

We summarized the findings based on the Cochrane Handbook for Outcomes [17]. We included grading to evaluate the quality of evidence based on the GRADE (Grading of Recommendations Assessment, Development and Evaluation) approach for each summary of findings table [18].

Results

Study Selection

**Figure 2:**
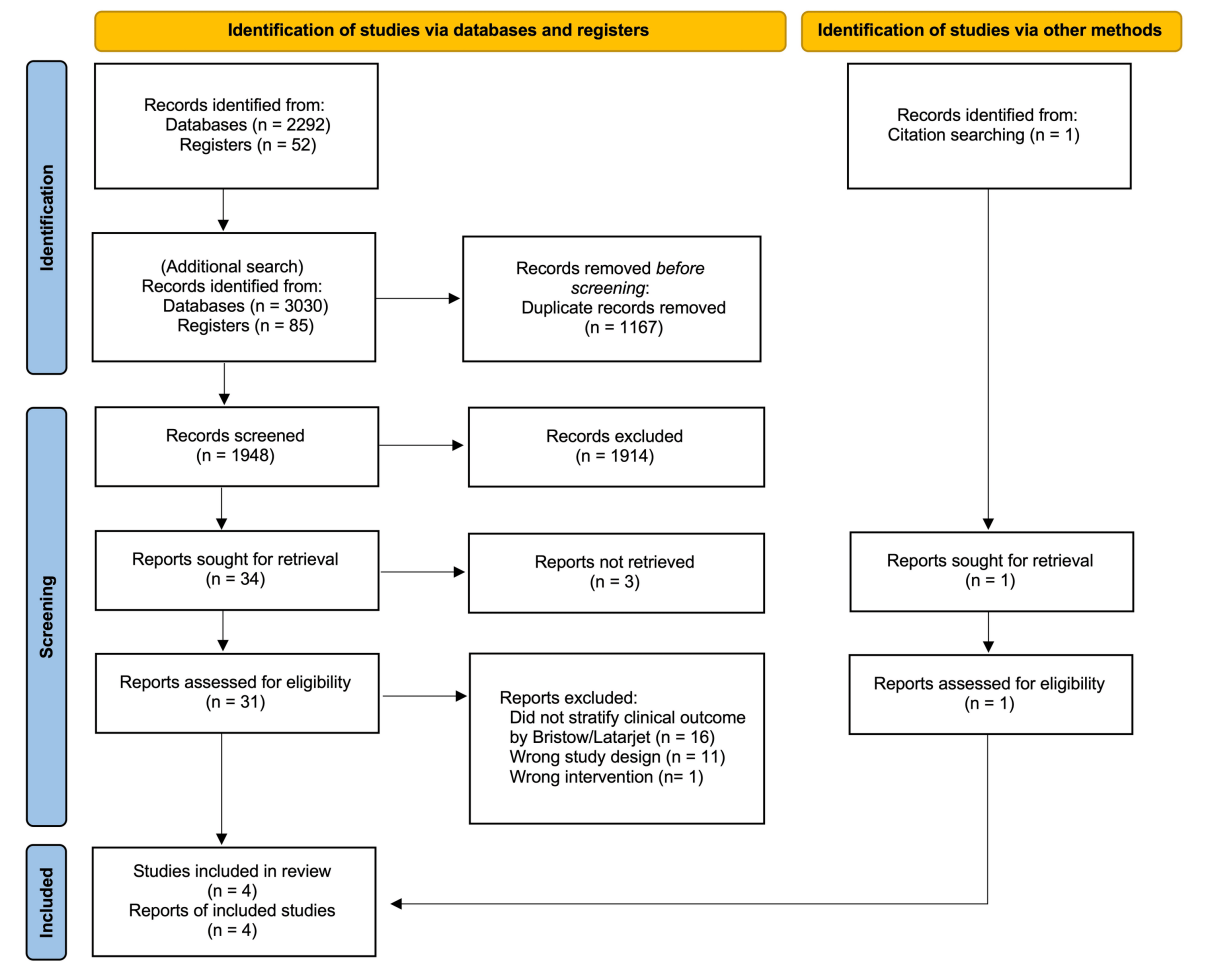
PRISMA flowchart of the articles included in the systematic review PRISMA: Preferred Reporting Items for Systematic Review and Meta-Analyses

In addition to the May 2023 search, we performed an additional search in March 2025. After removing duplicates, 1,948 records were screened for eligibility. After full-text review of 31 studies, four were selected (Figure 2) [10-12,19]. The studies that were excluded during the full-text screening stage are presented in the Appendices.

Study Characteristics

A total of 354 patients with 383 shoulders (211 shoulders treated with the Bristow technique, and 172 shoulders treated with the Latarjet technique) were included in the four studies [10-12,19].

The average age of patients who participated in these trials was 22.2 years (17.7-29.3 years). The average follow-up duration ranged from 32.4 to 73.5 months (Table 2).

**Table 2:** Characteristics of the included studies

Author	Year	Study design	Coracoid bone block procedure	Sample size, shoulders	Mean age, yr	Sex (M:F)	Sample extraction	Follow-up
Belangero et al. [10]	2021	Randomized controlled trial	Open Bristow	19	26.4	37:4	High-demand athletes	60 months
			Open Latarjet	22				
Shibuya et al. [11].	2021	Prospective cohort	Open Bristow	92	18.3 (17.5-19.1)	169:0	Rugby players	32.4 months
			Open Latarjet	77	19.1 (18.3-20.1)			
Tanaka et al. [12]	2022	Retrospective cohort	Open Bristow	66	18.4 (15-26)	101:0	Rugby Players	73.5 months
			Open Latarjet	35	17.7 (13-21)			63.5 months
Song et al. [19]	2023	Retrospective cohort	Arthroscopic Bristow	34	25.6	28:6	Contact-sport athletes	40.8 months
			Arthroscopic Latarjet	38	29.3	29:9		

Risk of Bias

**Figure 3:**
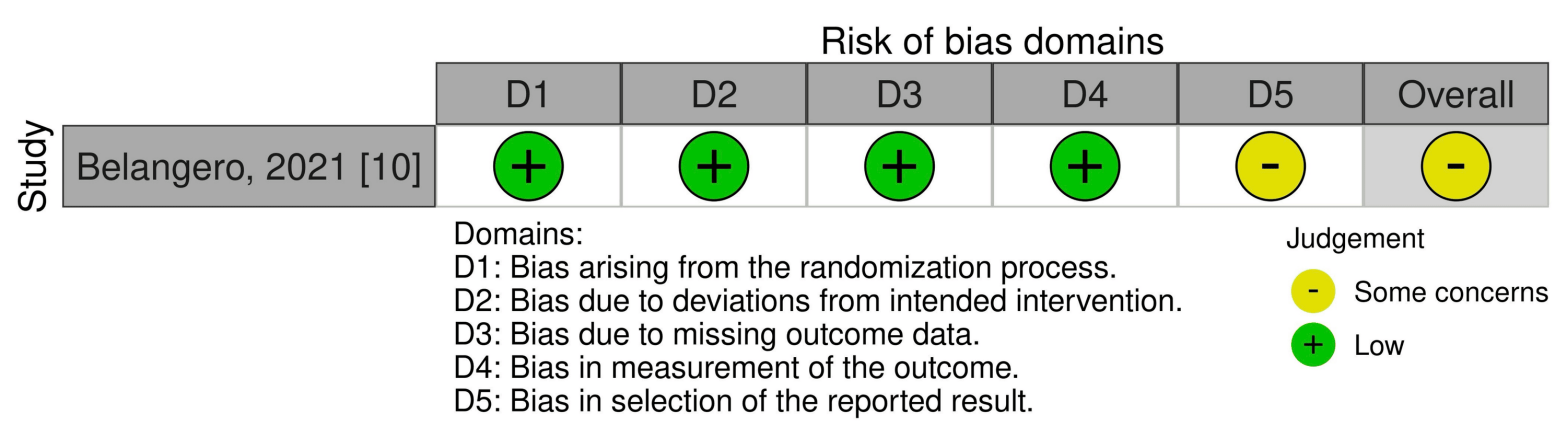
RoB 2 risk-of-bias assessment RoB 2: Risk of Bias 2

One RCT was assessed using RoB 2 and was considered to have some concerns (Figure 3).

**Figure 4:**
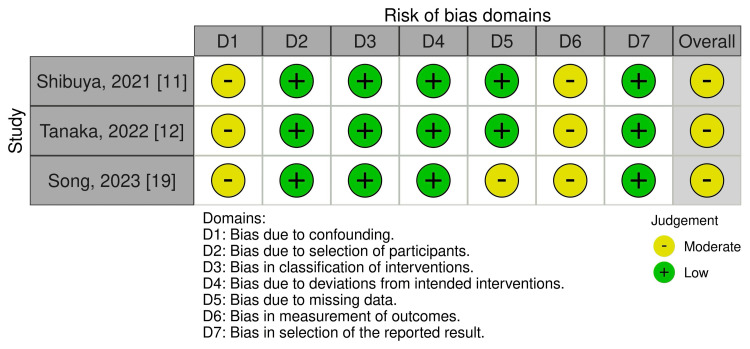
ROBINS-I risk-of-bias assessment ROBINS-I: Risk of Bias in Non-randomized Studies of Interventions

Three non-RCT studies were assessed using ROBINS-I, all of which indicated an overall moderate risk of bias (Figure 4).

Primary Outcomes

Redislocation rates were described in four studies, which included 383 shoulders (211 in the Bristow group and 172 in the Latarjet group) [10-12,19]. None of the patients in either the Bristow or Latarjet groups experienced redislocation in any study.

**Figure 5:**
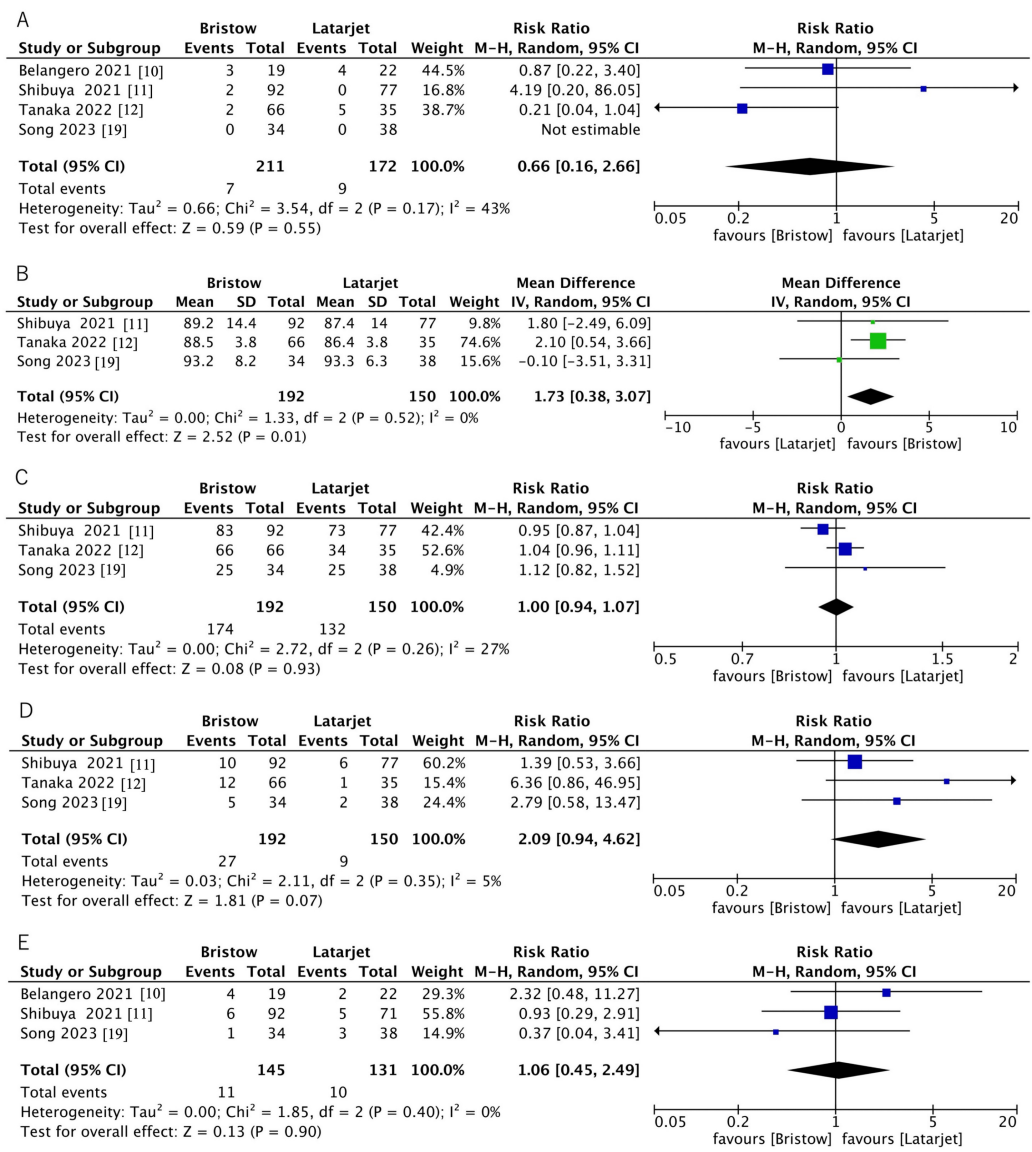
Forest plots of instability symptoms (A), Rowe score (B), return to sports (C), bone nonunion rates (D), and complications (E)

Instability symptoms were described in four studies, which included 383 shoulders (211 in the Bristow group and 172 in the Latarjet group) [10-12,19]. The definition of instability varied among the studies. Belangero et al. assessed instability only by the apprehension test, whereas Shibuya et al. and Tanaka et al. defined it as subluxations [10-12]. Song et al. reported instability using both subluxations and a positive apprehension test [19]. This analysis showed that the Bristow procedure may result in little to no difference in instability symptoms compared to the Latarjet procedure (RR, 0.66; 95% CI, 0.16-2.66; P = 0.55; low certainty of evidence) (Figure 5A; Table 3).

**Table 3:** Summary of findings CI: confidence interval; CoE: certainty of evidence; GRADE: Grading of Recommendations Assessment, Development and Evaluation; MD: mean difference; RCTs: randomized controlled trials; RR: risk ratio ^a^ Downgraded one level for inconsistency; ^b^ Downgraded one level for imprecision

Outcome	Participants (# studies)	Anticipated absolute effects (95% CI)		Risk with Bristow	CoE (GRADE)
		Risk with Latarjet	Risk with Bristow		
Redislocation	383 (1 RCTs, 3 Cohort)	0 per 1,000	0 per 1.000 (0 to 0)	Not estimable	⨁⨁⨁◯ Moderate^b^
Instability symptoms	383 (1 RCTs, 3 Cohort)	52 per 1,000	35 per 1.000 (8 to 139)	RR 0.66 (0.16 to 2.66)	⨁⨁◯◯ Low^a,b^
Rowe score	342 (3 Cohort)	-	MD 1.73 higher (0.38 higher to 3.07 higher)	-	⨁⨁⨁◯ Moderate^b^
Return to sports	342 (3 Cohort)	880 per 1,000	880 per 1.000 (827 to 942)	RR 1.00 (0.94 to 1.07)	⨁⨁◯◯ Low^a,b^
Bone nonunion	342 (3 Cohort)	60 per 1,000	125 per 1.000 (56 to 277)	RR 2.09 (0.94 to 4.62)	⨁⨁⨁◯ Moderate^b^
Bone resorption	Not pooled	Not pooled	Not pooled	Not pooled	-
Complications	276 (1 RCTs, 2 Cohort)	76 per 1,000	81 per 1.000 (34 to 190)	RR 1.06 (0.45 to 2.49)	⨁⨁◯◯ Low^a,b^

The Rowe score was described in three studies, which included 342 shoulders (192 in the Bristow group and 150 in the Latarjet group) [11,12,19]. The evidence suggests that the Bristow procedure results in a statistically significant difference in Rowe score compared to the Latarjet procedure (MD, 1.73; 95% CI, 0.38-3.07; P = 0.01; moderate certainty of evidence); however, this difference did not exceed the minimal clinically important difference (MCID) of 9.7 points for anterior shoulder instability, indicating that it is unlikely to be clinically meaningful (Figure 5B; Table 3).

Secondary Outcomes

Return to sports was described in three studies that included 342 shoulders (192 in the Bristow group and 150 in the Latarjet group) [11,12,19]. Song et al. evaluated return to sports using a structured four-level classification system (ranging from complete inability to resume sports to full return at the preinjury level) based on clinical assessment and patient-reported outcomes [19]. In contrast, Shibuya et al. and Tanaka et al. reported the timing of return to sports at four to five months after bone union confirmed by CT scans, but did not provide a standardized definition or classification system [11,12]. This analysis showed that the Bristow procedure may result in little to no difference in return to sports compared to the Latarjet procedure (RR, 1.00; 95% CI, 0.94-1.07; P = 0.93; low certainty of evidence) (Figure 5C; Table 3).

Bone nonunion was described in three studies, which included 342 shoulders (192 in the Bristow group and 150 in the Latarjet group) [11,12,19]. Tanaka et al. assessed bone union with CT scans and defined it as continuity of the trabecular bone between the coracoid process and glenoid [12]. Shibuya et al. assessed bone healing with CT scans at three months postoperatively [11]. Song et al. evaluated graft healing with the glenoid at three months and at final follow-up using the method adopted: nonunion was defined as a visible radiolucent line of less than 5 mm, while a radiolucent line greater than 5 mm indicated migration of the bone block; union was classified as bone healing at the graft-glenoid interface [19]. This analysis showed that the Bristow procedure probably results in little to no difference in bone nonunion compared to the Latarjet procedure (RR, 2.09; 95% CI, 0.94-4.62; P = 0.07; moderate certainty of evidence) (Figure 5D; Table 3).

Bone resorption was reported in three studies [10,12,19], but the evaluation methods varied. Belangero et al. assessed bone resorption using radiographs [10]. Song et al. evaluated bone resorption on the latest CT scan using a validated classification system with four grades: grade 0, no resorption; grade I, resorption only near the screw head; grade II, resorption of most of the graft; and grade III, total resorption [19]. Tanaka et al. also assessed bone resorption using CT scans and classified severity into four categories: none, mild, moderate, and severe [12]. Because of these heterogeneous definitions and imaging modalities, a pooled analysis of bone resorption could not be performed.

Complications were described in three studies, which included 276 shoulders (145 in the Bristow group and 131 in the Latarjet group) [10,11,19]. Reported complications encompassed surgical site infection, screw malposition or irritation, transient nerve palsy, and severe postoperative contracture requiring manipulation. This analysis showed that the Bristow procedure may result in little to no difference in complications compared to the Latarjet procedure (RR, 1.06; 95% CI, 0.45-2.49; P = 0.90; low certainty of evidence) (Figure 5E; Table 3).

Additional Analyses

We were unable to assess publication bias using funnel plots or Egger’s test because of the limited number of studies. Among the four included studies, three specifically recruited collision athletes (e.g., rugby or other contact sports), whereas one study included high-demand athletes, comprising both collision and non-collision types, without stratification of outcome data. Due to this inconsistency, subgroup analyses comparing collision versus non-collision athletes could not be performed. A sensitivity analysis for the Rowe score, excluding the study that used imputed statistics, yielded results inconsistent with those of the main analysis (MD, 1.54; 95% CI, -0.33-3.42; P = 0.11).

Discussion

This is the first study to compare the Bristow and Latarjet procedures for ASI by pairwise meta-analysis. The results showed little difference between the Bristow and Latarjet procedures in terms of redislocation rates, symptoms of instability, return to sports, bone nonunion and resorption, and complications; however, the Rowe score may be higher in the Bristow group. However, the difference was only 1.72 points, and considering that the MCID in anterior shoulder instability is at least 9.7 points for the Rowe score, the statistically significant difference in the Rowe score was not considered clinically important [20]. These results suggest that the Bristow and Latarjet procedures have little effect on postoperative clinical outcomes.

In 2019, Garcia et al. systematically reviewed the efficacy of the Bristow and Latarjet procedures for ASI [9]. In this study, 41 studies using the Bristow procedure reported a 1.00 ± 0.20% redislocation rate, and 18 studies using the Latarjet procedure reported a 2.13 ± 0.49% redislocation rate, which is a lower rate with the Bristow procedure than the Latarjet procedure. However, the review did not include RCTs or observational studies that directly compared the two surgical procedures, nor was a pairwise meta-analysis performed. In contrast, the present analysis included only studies that directly compared the Bristow and Latarjet procedures and found that no patients experienced redislocation after either of the two surgical procedures. However, this complete absence of re-dislocation events limited our ability to calculate pooled effect estimates for this outcome. As a result, the statistical power to detect meaningful differences between the procedures was reduced, and the clinical interpretation of their relative effectiveness in preventing redislocation remains uncertain. Nevertheless, a pairwise meta-analysis of instability symptoms suggested little to no difference in perceived instability between the Bristow and Latarjet procedures. However, moderate heterogeneity (I² = 43%) was observed in this outcome. Several factors may explain this inconsistency. First, although the included studies consistently evaluated instability symptoms, the specific definitions varied. Belangero et al. assessed instability solely by a positive apprehension test, Shibuya et al. and Tanaka et al. defined it as recurrent subluxations, and Song et al. combined both patient-reported subluxation episodes and a positive apprehension test [10-12,19]. Second, the study populations differed, with some studies limited to collision athletes such as rugby players, while others included mixed or high-performance athletes with variable recurrence risks. Taken together, these methodological and clinical differences are plausible contributors to the observed heterogeneity and should be considered when interpreting the pooled results.

The Bristow or Latarjet procedures are often applied to collision athletes [21,22]. Shibuya et al. reported no significant difference in the postoperative return-to-sports rates (90.2% for Bristow and 94.8% for Latarjet) for collision athletes using either surgical procedure [11]. Tanaka et al. reported high return-to-play rates for both surgical techniques (100% for Bristow, 96% for Latarjet), suggesting that both procedures were equally effective [12]. In contrast, a significantly higher percentage of collision athletes returned to the same level as before the injury with the Bristow (73.5%) than the Latarjet procedure (65.8%) [19]. The results of the meta-analysis showed that the Bristow and Latarjet procedures may result in little to no difference in return-to-sport rates. Therefore, the results suggest that the two surgical procedures are equally effective in terms of return to sports.

In this study, we analyzed the Rowe score, which was not reported in a previous review [9]. The results showed that the Bristow procedure probably results in little to no difference in the Rowe score compared to the Latarjet procedure (MD, 1.73; 95% CI, 0.38-3.07; P = 0.01). The difference in effect size between the two techniques was so small that, although Bristow appears to be more statistically valid, this difference is not clinically significant. Furthermore, a sensitivity analysis to examine the effect of substituting estimates for missing data found no significant difference in Rowe scores for the two methods (MD, 1.54; 95% CI, -0.33-3.42; P = 0.11). Taken together, these findings indicate that there is no clear difference between the two procedures in terms of the Rowe score, and the apparent effect may be driven by specific studies. However, the inconsistency observed between the primary analysis and the sensitivity analysis weakens the certainty of this outcome. This discrepancy reduces confidence in the robustness of our findings and highlights the need for additional high-quality randomized controlled trials to provide more reliable estimates of the comparative effectiveness of the Bristow and Latarjet procedures.

The condition of the osseous process graft is an important factor affecting the success of the Bristow and Latarjet procedures, with postoperative nonunion or resorption of the osseous process graft likely to result in revision surgery [23]. Two prior reviews reported nonunion rates of 9.4%-10.1% for the Bristow-Latarjet procedure [23,24]. Shibuya et al. compared the nonunion rates for both surgical methods (10.9% for the Bristow procedure and 7.8% for the Latarjet procedure) and reported no significant difference between the two groups [11]. Similarly, Song et al. reported no significant difference in the rate of nonunion (14.7% for the Bristow procedure and 5.3% for the Latarjet procedure) [19]. The results of the present study showed that, although the nonunion rate of the Latarjet procedure (6.9%) was lower than that of the Bristow procedure (11.9%), there was little to no difference between the two groups, similar to the results of a previous study.

Regarding the bone resorption of osseous process grafts, the bone resorption rate was reported to be approximately 70%-90% for the Latarjet procedure and 34.2% for the Bristow procedure [16,18,25,26]. In this meta-analysis, three studies examined bone resorption rates. Song et al. found bone resorption in 8.8% and 2.6% of patients who underwent the Bristow and Latarjet procedures, respectively, with no significant difference [19]. Belangero et al. found bone resorption in one case for each procedure, while Tanaka et al. found it in 6% for Bristow and 100% for Latarjet, but no statistical comparison was made [10,12]. Since these studies were evaluated by X-ray or CT scan, and there were differences in the severity classification of bone resorption between the studies, it was difficult to draw definitive conclusions from a comparison of the bone resorption rates of the two methods. The heterogeneity in bone resorption assessment methods represents a critical methodological issue in this field. The included studies used different imaging modalities (e.g., X-ray and CT scans) and applied inconsistent classification systems for defining and grading bone resorption. This variability not only prevented us from conducting a pooled quantitative analysis but also limited the comparability and interpretability of findings across studies. Such methodological inconsistency may have contributed to the wide variation in reported resorption rates, potentially obscuring the true incidence of this complication. Future studies should adopt standardized imaging protocols and uniform classification criteria to enable more reliable comparisons and synthesis of evidence.

The incidence of postoperative complications of the Bristow-Latarjet procedure in collision athletes ranges from 0.8% to 19.2%, with postoperative infection, postoperative coracoid fracture, severe pain, screw loosening, and screw breakage noted [27]. Three studies reported complications: nerve palsy, postoperative infection, severe contracture, coracoid process avulsion, and screw malpositioning [10,11,19]. Two studies reported complication rates ranging from 2.9% to 21.0% for the Bristow procedure and 7.8% to 9.0% for the Latarjet procedure [10,19]. Shibuya et al. compared the complication rates of both surgical procedures and reported no significant differences between the Bristow (6.5%) and Latarjet (7.0%) procedures [11]. Similarly, the analysis in this study showed that the Bristow (7.5%) and Latarjet procedures (7.6%) may result in little to no difference in complications [10,11,19].

The limitations of this study are as follows. First, the analysis included observational studies as well as RCTs comparing the Bristow and Latarjet procedures. Observational studies inherently have a greater risk of bias because of the non-random allocation of treatments, which limits causal inference and may introduce selection bias. Although some reports suggest that well-designed observational studies in surgical fields can yield results comparable to RCTs [28,29], the predominance of observational evidence in the present analysis remains a major limitation, underscoring the need for further high-quality RCTs to validate the evidence. Second, the participants in these studies were athletes from a variety of sports competitions. Three of the four studies included in this review were conducted with contact athletes, while the remaining study was conducted with high-activity athletes (high-demand athletes) such as soccer, handball, and volleyball players [10-12,19]. Subgroup analyses between collision and non-collision athletes were not performed because of a lack of data. Third, we were unable to assess publication bias using funnel plots or Egger’s test because the number of included studies was insufficient. Therefore, the possibility of publication bias cannot be excluded, which may limit the generalizability of our findings. Fourth, all included studies reported no redislocations in either surgical group. This absence of events limited our ability to perform quantitative analysis for this outcome, thereby reducing the statistical power and interpretability of our findings regarding redislocation. Fifth, the results of this study are based on moderate to low certainty of evidence. The main factor contributing to the low certainty of evidence for many of the outcomes in this study was imprecision. The insufficient sample size and number of events were conspicuous, hindering accurate effect estimates. To address this imprecision issue, ensuring a sufficient sample size in future studies may allow a more accurate capture of the true magnitude of the effects. However, the strength of this study is that it directly compared the clinical outcomes of the Bristow and Latarjet procedures for ASI using a pairwise meta-analysis, providing new insights.

## Conclusions

In this study, a pairwise meta-analysis of previously reported clinical outcomes of the Bristow and Latarjet procedures for ASI was conducted and demonstrated little to no difference in redislocation rate, instability symptoms, Rowe score, return to sports, bone nonunion, and complications between the two procedures. However, because the included studies involved athletes from diverse sports, including both collision and non-collision types, without stratification, these findings should be interpreted with caution and may not be fully generalizable across all athlete populations.

## Appendices

**Table 4:** List of studies excluded from this review and reasons for exclusion

Reason for exclusion	References
Did not stratify clinical outcome by Bristow/Latarget	Hovelius L, Körner L, Lundberg B, Akermark C, Herberts P, Wredmark T, Berg E. The coracoid transfer for recurrent dislocation of the shoulder. Technical aspects of the Bristow-Latarjet procedure. The Journal of Bone and Joint Surgery. 1983. 65(7): p. 926-934.
	Carol EJ, Falke LM, Kortmann JH, Roeffen JF, van Acker PA. Bristow-Latarjet repair for recurrent anterior shoulder instability; an eight-year study. The Netherlands Journal of Surgery, 1985. 37(4): p. 109-113.
	Hovelius LK, Sandström BC, Rösmark DL, Saebö M, Sundgren KH, Malmqvist BG. Long-term results with the Bankart and Bristow-Latarjet procedures: recurrent shoulder instability and arthropathy. Journal of Shoulder and Elbow Surgery, 2001. 10(5): p. 445-452.
	Hovelius L, Sandström B, Sundgren K, Saebö M. One hundred eighteen Bristow-Latarjet repairs for recurrent anterior dislocation of the shoulder prospectively followed for fifteen years: Study I - Clinical results. Journal of Shoulder and Elbow Surgery, 2004. 13(5): p. 509-516.
	Spoor AB, de Waal Malefijt J. Long-term results and arthropathy following the modified Bristow-Latarjet procedure. International Orthopaedics, 2005. 29(5): p. 265-7.
	Hovelius L, Sandström B, Saebö M. One hundred eighteen Bristow-Latarjet repairs for recurrent anterior dislocation of the shoulder prospectively followed for fifteen years: study II-the evolution of dislocation arthropathy. Journal of Shoulder and Elbow Surgery, 2006. 15(3): p. 279-289.
	Emami MJ, Solooki S, Meshksari Z, Vosoughi AR. The effect of open Bristow-Latarjet procedure for anterior shoulder instability: a 10-year study. Musculoskeletal Surgery, 2011. 95(3): p. 231-235.
	Hovelius L, Vikerfors O, Olofsson A, Svensson O, Rahme Hans. Bristow-Latarjet and Bankart: a comparative study of shoulder stabilization in 185 shoulders during a seventeen-year follow-up. Journal of Shoulder and Elbow Surgery, 2011. 20(7): p. 1095-1101.
	Gordins V, Hovelius L, Sandström B, Rahme H, Bergström U. Risk of arthropathy after the Bristow-Latarjet repair: A radiologic and clinical thirty-three to thirty-five years of follow-up of thirty-one shoulders. Journal of Shoulder and Elbow Surgery, 2015. 24(5): p. 691-699.
	Ruci V, Duni A, Cake A, Ruci D, Ruci J. Bristow-Latarjet Technique: Still a Very Successful Surgery for Anterior Glenohumeral Instability - A Forty Year One Clinic Experience. Open access Macedonian Journal of Medical Sciences, 2015. 3(2): p. 310-4.
	Sakeb N, Islam MA, Jannat SN. Outcome of modified Bristow-Laterjet procedure in post-traumatic recurrent anterior shoulder dislocation in young population. Mymensingh Medical Journal : MMJ, 2015. 24(1): p. 74-83.
	Beranger JS, Klouche S, Bauer T, Demoures T, Hardy P. Anterior shoulder stabilization by Bristow-Latarjet procedure in athletes: return-to-sport and functional outcomes at minimum 2-year follow-up. European Journal of Orthopaedic Surgery & Traumatology : Orthopedie Traumatologie, 2016. 26(3): p. 277-282.
	Moroder P, Stefanitsch V, Auffarth A, Matis N, Resch H, Plachel F. Treatment of recurrent anterior shoulder instability with the Latarjet or Bristow procedure in older patients. Journal of Shoulder and Elbow Surgery, 2018. 27(5): p. 824-830.
	Filho JG, Leite MC, Borges ACW, de Souza GT, do Prado OF. Clinical and Radiographic Evaluation of Patients Operated by the Bristow-Latarjet Technique with a Minimum Follow-Up of 20 Years. Revista Brasileira De Ortopedia, 2020. 55(4): p. 455-462.
	Yang YL, Li QH, Zhang Q, Jia HL, Wang BM, Dong JL, Zhou DS, Wang XM, Li LX. Treatment of Chronic Anterior Shoulder Dislocation by Coracoid Osteotomy with or without Bristow-Latarjet Procedure. Orthopaedic Surgery, 2020. 12(5): p. 1478-1488.
	Eberlin CT, Varady NH, Kucharik MP, Naessig SA, Best MJ, Martin SD. Comparison of perioperative complications following surgical treatment of shoulder instability. JSES International, 2022. 6(3): p. 355-361.
Wrong study design	Jalovaara P, Niinimaki T, Ramo J, Lindholm RV. Coracoid tendon transposition a.m. Bristow-Latarjet. Annales Chirurgiae et Gynaecologiae, 1988. 77(3): p. 103-107.
	Dossim A, Abalo A, Dosseh E, Songne B, Ayite A, Gnandi-Pio F. Bristow-Latarjet repairs for anterior instability of the shoulder: Clinical and radiographic results at mean 8.2 years follow-up. Chirurgie de la Main, 2008. 27(1): p. 26-30.
	Doursounian L, Debet-Mejean A, Chetboun A, Nourissat G. Bristow-Latarjet procedure with specific instrumentation: Study of 34 cases. International Orthopaedics, 2009. 33(4): p. 1031-1036.
	Hovelius L, Sandström B, Olofsson A, Svensson O, Rahme H. The effect of capsular repair, bone block healing, and position on the results of the Bristow-Latarjet procedure (study III): long-term follow-up in 319 shoulders. Journal of Shoulder and Elbow Surgery, 2012. 21(5): p. 647-60.
	Paladini P, Merolla G, De Santis E, Campi F, Porcellini G. Long-term subscapularis strength assessment after Bristow-Latarjet procedure: isometric study. Journal of Shoulder and Elbow Surgery, 2012. 21(1): p. 42-47.
	Jiménez I, Marcos-García A, Medina J, Muratore-Moreno G, Caballero-Martel J. Bristow-Latarjet Technique for anterior glenohumeral instability. Acta Ortopedica Mexicana, 2016. 30(6): p. 291-295.
	Tang K, Zhang H. The mini-open modified bristow-latarjet procedure for anterior shoulder instability: A 15-year study on the results of 90 cases. Arthroscopy - Journal of Arthroscopic and Related Surgery, 2017. 33(10): p. e162-e163.
	Lavoué V, Gendre P, Saliken D, Brassac A, Boileau P. The Role of Arthroscopic Soft Tissue Reconstruction for Failed Bristow-Latarjet Procedure. Arthroscopy - Journal of Arthroscopic and Related Surgery, 2019. 35(9): p. 2581-2588.
	Ribeiro LM, Lara PHS, Pochini AC, Andreoli CV, Belangero PS, Ejnisman B. Isokinetic Evaluation of the Shoulder After Bristow/Latarjet Surgical Procedure in Athletes. Revista Brasileira De Ortopedia, 2021. 57(1): p. 128-135.
	Sano H, Komatsuda T, Abe H, Ozawa H, Kumagai J, Yokobori T. Intra-articular biomechanical environment following modified Bristow and Latarjet procedures in shoulders with large glenoid defects: relationship with postoperative complications. Journal of Shoulder and Elbow Surgery, 2021. 30(10): p. 2260-2269.
	Zhang S, Zhang M, Shao Z, Cui G. Research progress in biomechanics of Bristow-Latarjet procedure for anterior shoulder dislocation. Zhongguo Xiu Fu Chong Jian Wai Ke Za Zhi = Zhongguo Xiufu Chongjian Waike Zazhi = Chinese Journal of Reparative and Reconstructive Surgery, 2023. 37(5): p. 518-525.
Wrong intervention	Pihlajamäki H, Böstman O, Rokkanen P. A biodegradable expansion plug for fixation of the coracoid bone block in the Bristow-Latarjet operation. International Orthopaedics, 1994. 18(2): p. 66-71.
